# 

**Published:** 1902-08

**Authors:** 


### CONTRIBUTORS TO NEW SERIES VOLUME XLII.

### (Whole Number Volume LVIII.)

### AUGUST, 1902 – JULY, 1903.

| | |
|---|---|
| Auerbach, M. A. | New York |
| Barnes, A. C. | Philadelphia |
| Bayliss, A. W. | Buffalo |
| Beahan, A. L. | Canandaigua |
| Benedict, A. L. | Buffalo |
| Benninghoff, G. E. | Bradford |
| Bissell, William G. | Buffalo |
| Burke, Joseph | Buffalo |
| Burnham, N. L. | Buffalo |
| Burrows, Lorenzo, Jr. | Buffalo |
| Button, C. A. | Holland |
| Clark, Joseph C. | Olean |
| Colton, A. J. | Buffalo |
| Congdon, Charles E. | Buffalo |
| Creveling, J. P. | Auburn |
| Crothers, T. D. | Hartford |
| Cumston, Charles Greene | Boston |
| Daggett, Byron H. | Buffalo |
| Davie, Carey D. | Salamanca |
| Dearden, J. E. | New York |
| Diehl, Alfred E. | Buffalo |
| Dorr, L. Bradley | Buffalo |
| Dowd, J. Henry | Buffalo |
| Drake, W. F. | New York |
| Dunham, Sydney A. | Buffalo |
| Forsyth, Edgar A. | Buffalo |
| Fowler, George Ryerson | Brooklyn |
| Fronczak, Francis E. | Buffalo |
| Frye, Maud J. | Buffalo |
| Gaylord, Harvey R. | Buffalo |
| Goldberg, Sigmund | Buffalo |
| Grove, Benjamin H. | Buffalo |
| Hall, A. L. | Fulton |
| Haas, Leopold F. W. | New York |
| Heffron, John L. | Syracuse |
| Heuman, C. C. | New York |
| Hopkins, Henry Reed | Buffalo |
| Jack, George N. | Depew |

Kendall, W. P. .......... Buffalo
Krauss, William C. .......... Buffalo
Long, Eli H. .......... Buffalo
Luedeking, Robert .......... Saint Louis
Lyon, Irving Phillips .......... Buffalo
Macpherson, J. D. .......... Akron
Mansperger, William H. .......... Buffalo
McGuire, Francis W. .......... Buffalo
McKee, Thomas H. .......... Buffalo
Miller, John A. .......... Buffalo
Myers, J. F. .......... Sodus
Park, Roswell .......... Buffalo
Peterson, Frederick .......... New York
Phelps, William C. .......... Buffalo
Potter, William Warren .......... Buffalo
Putnam, James Wright .......... Buffalo
Raub, J. F. .......... Washington
Reed, Charles A. L. .......... Cincinnati
Reisman, S. C. .......... New York
Ruffner, E. L. .......... United States Army, Iloilo, P. I.
Russell, William L. .......... Willard
Ryerson G. Sterling .......... Toronto
Satterlee, Richard H. .......... Buffalo
Saxe, G. A. de Santos .......... New York
Snow, Irving M. .......... Buffalo
Snow, Sargent F. .......... Syracuse
Stephenson, F. H. .......... Syracuse
Von Ruck, Silvio .......... Asheville
Weiss, Ludwig .......... New York
Wende, Grover W. .......... Buffalo
Westinghouse, G. H. .......... Buffalo
Whipple, Electa B. .......... Buffalo
Williams, Henry T. .......... Rochester
Wilson, Nelson W. .......... Buffalo
Woehnert, A. E. .......... Buffalo
Zimmer, John .......... Rochester

Alumni Association University of Buffalo.
American Association of Obstetricians and Gynecologists.
Buffalo Academy of Medicine.
Medical Association of Central New York.
Medical Society of the County of Erie.

### BOOKS RECEIVED.

A Manual of Instruction in the Principles of Prompt Aid to the Injured. Including a Chapter on Hygiene and the Drill Regulations for the Hospital Corps, U.S.A. Designed for Military and Civil Use. By Alvah H. Doty, M. D. Health Officer of the Port of New York. Fourth edition, revised and enlarged. New York: D. Appleton & Co. 1902.

Mother and Child. By Edward F. Davis, A. M., M. D., Professor of Obstetrics in the Jefferson Medical College; Professor of Obstetrics and Diseases of Infancy in the Philadelphia Polyclinic. Duodecimo, pp. 264. Philadelphia: J. B. Lippincott Co. 1902. ($1.50)

A Treatise on Diseases of the Skin. For the use of Advanced Students and Practitioners. By Henry W. Stelwagon, M. D., Ph. D., Clinical Professor of Dermatology, Jefferson Medical College and Woman's Medical College, Philadelphia; Dermatologist to the Howard and Philadelphia Hospitals. Handsome octavo of 1125 pages, with 220 text-illustrations, and 26 full-page lithographic and half-tone plates. Philadelphia and London: W. B. Saunders & Co. 1902. (Cloth, $6.00 net; Sheep or Half-Morocco, $7.00 net.)

Saunders's Medical Hand-Atlases. Atlas and Epitomé of Abdominal Hernias, By Privat docent Dr. Georg Sultan, of Göttingen. Edited, with additions, by William B. Coley, M. D., Clinical Lecturer on Surgery, Columbia University (College of Physicians and Surgeons) With 119 illustrations, 36 of them in colors, and 277 pages of text. Philadelphia and London; W. B. Saunders & Co., 1902. [Cloth, $3.00 net.]

Medical and Surgical Report of the Presbyterian Hospital, New York. Volume V. January, 1902. Edited by Drs. Andrew J. McCosh and W. Gilman Thompson.

The Roller Bandage. By William Barton Hopkins, surgeon to Pennsylvania Hospital and to the Orthopedic Hospital and Infirmary for Nervous Diseases. 12mo, pp. 178. Fifth edition, revised. Philadelphia: J. B. Lippincott Company. 1902. [Price, $1 50.)

Vaughan and Novy on Cellular Toxins. A treatise on Cellular Toxins, or the Chemical Factors in the Causation of Disease. Bv Victor C. Vaughan, Ph.D., M.D.,Professor of Hygiene and Physiological Chemistry, and Frederick G. Novy, M. D., Junior Professor of Hygiene and Physiological Chemistry in the University of Michigan. New (4th) edition, revised and enlarged. In one 8vo volume of 480 pages, with six illustrations. Lea Brothers & Co., Philadelphia and New York. 1902. [$3.00 net.]

The Practical Medicine Series of Year Books. Ten volumes. Issued monthly under the general editorial charge of Gustavus P. Head, M. D., Professor of Laryngology and Rhinology in the Chicago Post-Graduate Medical School. Volume VII. Materia Medica and Therapeutics; Preventive Medicine; Climatology; Forensic Medicine. By George F. Butler, Ph.G., M.D., Henry B. Favill, A.B., M.D., Norman Bridge, A.B., M.D., Harold N. Moyer, M.D. 12mo, pp. 279. Chicago: The Year Book Publishers. 1902. [$1.50. Entire series, $7.50.]

The Johns Hopkins Hospital Reports. Volume X., Nos. 3, 4 and 5. Baltimore: The Johns Hopkins Press. 1902.

